# Anæsthesia and Anæsthetics

**Published:** 1892-09

**Authors:** S. J. Hayes


AMERICAN JOURNAL

—OF—

DENTAL SCIENCE.

VOL. XXVI. BALTIMORE, SEPTEMBER, 1892. NO. 5.

ARTICLE I.

### ANÆSTHESIA AND ANÆSTHETICS.

BY DR. S. J. HAYES.

**An address delivered to the Cincinnati Medical Society.**

It is to be very much regretted that there is no other science connected with the healing art, that is so little understood, or of which there has been so little known, as the science of Anæsthesia. True, there has been no *great dearth* in writing up the subject, but what has been written, is mainly historical, and principally a rehash of what others have said or done, without a proper investigation into cause and effect. And yet, no one dare question but that to *relieve* from the agonies of pain and to render comparatively easy and safe *delicate* and *intricate* surgical operations is certainly the most *gracious boon* that can possibly be conferred by the Medical and Dental professions upon suffering humanity. That which lays at the very foundation of the study of this science, is a knowledge of the constitution of the nervous system, its *organic structure* and the laws by which it is regulated and governed. This is most certainly manifest, in that we *must* deal *primarily* and *all the time* with this most intricate and yet marvelously delicate organic structure, in which there is such a wonderful range of difference, the powers of endurance so *varied* and so widely, and yet, strangely differing, that it is simply *incomprehensible* and *inexplicable* with our limited knowledge of this structure. Yet, we may know something of its constituency, the laws in relation thereto and the functions of life, and the more we know, the better we are qualified to prepare and adapt agencies to produce anæsthesia safely, pleasantly, effectually and without unpleasant after-results. I shall not now attempt to depict by diagram or chart the organic nervous system, leaving that to other and more capable hands, but I desire to call your attention to some of the laws relative to its constitution and functions, which are of *most vital importance* in relation to anæsthesia. For, mark you, anæsthesia is *not* simply a condition in which there is an *absence of sensation* as it is usually defined, but a condition in which the force and functions of life are in continuance, under modification, induced by medico-chemical agencies. And just here, I prefer to have it understood, that while I am speaking didactically, I do not speak authoritatively, and much prefer to express my own thoughts rather than the thoughts of others, more beautifully expressed, knowing full well that you will be capable of separating and gathering in the wheat and rejecting the chaff. Authority is a very good thing, and will assist in developing the arts and sciences when *right*, but when *wrong*, it is a very great evil, and materially retards the development.

While oxygen is the force of life, or that out of which *vital force* or nerve-vital fluid or animo-electricity, is generated by the pulmonary glands, yet it is not life itself. Life is an imponderable agent located in the organic nervous system which requires oxygen to make manifest the operations which characterize life, respiration, circulation and animal heat. It is believed, that life is imparted at the instant the semen is emitted. Life may continue dormant for a number of days, as will be seen by persons laying in a trance, apparently lifeless, yet again, having resumed the functions of life. Persons taken from out of the water, to all appearance lifeless, and without any manifestation of functional life, have had respiration, circulation and heat established. A frog may be frozen to represent an icicle, and yet when thawed, it will leap about sprightly. Thus it will be seen that while we may not be able to describe life for lack of its physical characteristics, we must recognize it as an *independent power* or agent capable of holding possession of its abode in the organic nervous system for a limited time, independently or without the presence of either the oxygen, or the blood. This is fully established by the fact that life will remain in the organic nervous system quite a while after all the blood, and consequently oxygen has been removed. Life being an invisible imponderable agent, or power, under whose guidance or force all the complicated organizations of man carry on their functions, and being located in the organic nervous system, only requires oxygen and blood to perform all the wonderful phenomena of life, and display its wisdom.

The heart and lungs are regulated by the cardiac and pulmonary ganglions and the pulmonary glands, secrete animal electricity or *nervo-vital* fluid. This is demonstrated beautifully in the evolution of electricity by the torpedo. That the pulmonary organic glands are capable of generating or giving off animal electricity or vital fluid by the entrance of air into the lungs, and its passage through the glands and union with venous blood, is clearly manifest in this peculiar species of the finny tribe. The partitions of the electrical column are covered with a fine net-work of arteries, veins and nerves. They are very vascular, sending inward from the circumference, all around on each partition, small arteries which anastomoze upon it, and passing also, from one to another, unite with the vessels of the adjacent partition. Goldstream says: The nerves of the electrical organs of the Gymnotus are derived from the spinal marrow alone. They are very large and numerous, and are divided into very fine twigs on the cells of the organs. There is a wonderful similarity in a section of the electric organ of the Torpedo and Gymnotus and a section of the lung. The sections of both organs are furnished with arteries, veins, nerves and cells. Both these organs are amply supplied with animal nerves derived from the spinal chord, and therefore the medulla is continuously supplied with this nerve-vital fluid, and is the seat or reservoir of *vital force.* This vital force, or nervo-vital fluid, is sent out from the medulla through the nerves at the behest of life itself.

Thus it will be seen, that by the union of oxygen with the pulmonary glands and venous blood, the functions of life are made manifest as characterized by respiration, circulation and animal heat. Therefore, it follows, that whatever has a tendency to weaken or strengthen the action of the heart, will accordingly effect the temperature of the body; and also, whatever depresses or excites the nervous system, weaken or strengthen respiration, and the functions of the heart, that being the result of the evolution of electricity or nervo-vital fluid by the pulmonary glands, and the union of oxygen with the blood, which latter can only be effected by the evolution of animal electricity as evolved by these glands and the cerebral glands of the brain. Consequently, as you suspend or cut off the supply of free oxygen to the lungs, the pulmonary glands cease to generate and furnish *vital force*, both to the nervous system and to the blood. The *safety* and *continuance* of life depends upon a sufficient supply of *free oxygen* to furnish sufficient vital force to sustain the functions of life. If it be true, that anæsthesia is *only* a condition in which there is an absence of sensation as it is usually defined, then either asphyxia, syncope, paralysis or death would be anæsthesia, as in these conditions there is an absence of sensation. Yet, no sane, well-informed man will ever want either of these physical conditions for a safe surgical operation. Words are employed to represent ideas, and they should represent them so clearly that there be no confounding nor leading astray by their use. I should say that anæsthesia is a physical condition differing *radically* from every other, in this, that in this condition the forces and functions of life, must be in continuance under modification—modification induced by medico-chemical agencies—suitable narcotics, whereby an absence of sensation is attained. The force of life, being oxygen in a *free* state without which *you* cannot produce anæsthesia, it is the all important element in your anæsthetic. Free oxygen is oxygen, *not chemically combined* with any other chemical element, but simply in a state of mixture as found in the atmosphere. The moment you combine it chemically as in Nitrous Oxide Gas (N2O) it loses its properties and functions. This is in accordance with the fundamental law of chemistry which is more binding than the laws of the Medes and Persians, because it is founded in nature itself and is as eternal. Consequently, agencies that do not contain free oxygen, such as the numerous preparations from methelic or ethelic alcohol, as Ether, Chloroform, etc., are not anæsthetics for the reason that they do not contain oxygen in a free state, but are simply suitable narcotics producing exciting sedative, soporific or iodide effects, depending upon the state of administration. Therefore, it will be readily seen, that you make your anæsthetic when you use any of these narcotics and that the narcotic preparation is simply a modifier and the subordinate element in your anæsthetic. You may modify by a super quantity of oxygen in a free state, which you have by rapid inhalation and which produces a toxicological effect upon the nerve centres and by which you may produce anæsthesia in some persons, sufficient to perform a minor surgical operation without incurring pain. However, your suitable narcotic is not only a convenience, but very necessary as a subordinate element in your anæsthetic. Yet, you must have sufficient free oxygen to furnish the red corpuscles in the blood and to sustain life without which you cannot have anæsthesia but will have asphyxia and you may have death. Asphyxia can *only* be produced by suspension, abrogation or cutting off of the force of life (oxygen in a free state) and then when you do not have sufficient force (free oxygen) to eliminate the narcotic from the blood and system, your patient sinks and gradually looses the functions of life and then all the powers of earth cannot resuscitate. Unpardonable and outrageous errors have been committed by many writers on the subject of "Anæsthetics and Artificial Anæsthesia" in confounding mere asphyxiating agents with true anæsthetics, and in not recognizing and representing the marked difference both in their properties and functions as well as in their effects.

Asphyxia is the natural and legitimate result of depriving the blood of oxygen in its simple and chemically unconnected form. Anæsthesia is a state of insensibility induced ordinarily by narcotizing the patient with a suitable narcotic while sustaining the force and functions of life, with this life-giving principle. Therefore, while insensibility is an attribute of asphyxia as well as of anæsthesia, yet they are radically different and diametrically opposite in their tendencies and results. The former may be properly designated as "the first step in at death's door," while the latter is the happy state of *insensibility* with the possession of the forces and functions of life. The failure of Nitrous Oxide ($N_2O$) Carbonic Oxide ($CO$) or Carbonic Acid ($CO_2$) or any other similar inert gas to sustain respiration and life, is not attributable to a lack of oxygen in them as they contain a greater quantity of oxygen than the atmosphere, but that the oxygen and nitrogen or carbon in the gases are in *chemical union* and that the affinity of the oxygen for the other gas, is greater or stronger than the affinity of the blood for the oxygen.

and consequently the blood cannot appropriate the oxygen and, therefore, these gases fail to support life. In the atmosphere (N4 O) these gases are in a state of admixture, not in chemical union and therefore the oxygen readily enters the blood to sustain life. And since pure undiluted oxygen is too irritating and exciting to sustain life without detriment and since as it is found diluted with nitrogen in the atmosphere, it is readily appropriated and meets the wants of life perfectly, and since when found in any other condition or combination and appropriated, it always endangers health and life, the use of Nitrous Oxide, Carbonic Acid or any other asphyxiating agent for the pretended purposes of producing anæsthesia, should be prohibited by appropriate legislation. In fact, these agencies antagonize life from the first inspiration. And a cat or a dog, or a man will cross over the death line when the exclusive use of Nitrous Oxide Gas or any other asphyxiating agent, has so weakened the affinity of the blood for oxygen that it cannot appropriate the oxygen when the air is admitted to the lungs. And although, thousands have taken this so called "Laughing Gas" without apparent injury, yet, it antagonizes life from the very first inspiration and no man, however strong, can be deprived of sensation by the loss of *vital force* without some injury in some way to his nervous system. The loss of *vital force* and *neural energy* in many have resulted in consumption and insanity, or both. Hence, the use of asphyxiating agencies, as Dr. Richardson says, "should be prohibited by law." But when an anæsthetic is inhaled, the narcotic element passes into the lungs with the oxygen, the narcotic is thus communicated on the union of the oxygen with the organic glands and simultaneously into the glands and the organic ganglions and into the venous blood. The narcotic agent is thus conveyed into the nerve tubules which enter into the organization of the ganglions by their connection with the organic glands and the vascular membranes which surround the ganglions.

Thus, the formation of the ganglions, are interfered with and the superior central ganglion and the cerebral glands are placed in a *quiescent state* and cease giving off this *nervo vital fluid* to stimulate the nerve fibers of the brain and the mind ceases to act, or acts *feebly* and sleep is the result. The narcotic element is also communicated to the external, middle and internal coats of the arteries by the arterial blood. It is still further conveyed to the muscular fibres by their connection with the capilary arteries, causing their relaxation and ultimately interfering with the action of the pulmonary glands which will only occur when you have an over-percentage of the narcotic element in your anæsthetic.

Before a person is fully anæsthetized, he gets apparently drunk. This is caused by the confused action of the brain, consequent upon the irregular and interrupted secretion of the nervo-vital fluid by the cerebral glands.

Having described the channel through which the chemical and mechanical effects of anæsthetics, are produced upon the nerve centres which may be denominated the secondary or remote effects; I will now speak of the immediate or primary effects upon the nerve centres, the nervous system and their relations to the functions of life. The axiom—"never a result without a cause" must be borne in mind. When the statistical reports of deaths from the use of chloroform, ether, etc., show that the most of the deaths have occurred in the first stage and in the hot season of the year, we inquire for the cause. In so doing, we ascertain that the anæsthetics are introduced into the system very much as electricity is introduced, namely, through the *sensory nerves*. The pneumo-gastric nerve extending into the lungs, and stomach, is principally a sensory nerve. The sensory nerves communicate from without to within the system, or from the periphery to the nerve centres, and the *vassomotor nerves* from within to without, or from the nerve centres to the periphery. There is a circulation independent (and yet not independent) of arterial and venous circulation. The circulation of a substance within the nerves, which for want of a better name, we call *nervo-vita fluid.* This is the medium of communicating intelligence to the nerve centres and power to the physical organs, whereby they are enabled to perform their functions, and without which there can be no *neural force* nor *physical power.* Consequently, not a function of life can be performed when this circulation stops. As the functions of the lungs and the heart, as well as every other organ, are performed at the demand of the medulla and the behest of life, when the circulation of this vital fluid in the medulla is suddenly arrested by the abrupt introduction of an over quantity of electricity, or an over percentage of a powerful narcotic, such as chloroform, or by a sudden action of the mind in an extremely delicately, nervously constituted person, you have *concussion, revulsion* in neural circulation and paralyzation. There are two agencies operating upon and through neural circulation and neural force—the one internal, the other external. The former, we denominate intellect, the latter, *chemical* and mechanical and these two agencies produce similar effects.

By a sudden, startling action of the mind as by the sight of blood or fright or sudden death, revulsion in neural circulation in the sympathetic pneumo-gastric nerve in the stomach, is produced and nausea is the result; a little later on you have its *reflex* in the medulla a partial paralysis, and you have syncope. Or the action of the mind may be so quick and *powerful* that a terrible concussion, revulsion and paralyzation is the result, sometimes producing death. The latter condition is frequently produced by the sight of a revolting death, or by the sudden breaking of *very sad news* to an extremely delicately nervously constituted person, such as the sudden and terrible death of a very near and dear friend. This is a sudden *arrestation* of the circulation of the nervo-vital fluid in the medulla, and when this occurs every function of life suddenly ceases. The very same effects are produced by the external agencies. The sudden introduction of too great a volume of electricity or an over percentage of the narcotic element in an anæsthetic or *severe pain* (which is mechanical) will produce nausea, syncope, paralysis or death. If you have the functions of life cease at one, two, three or four inhalations of chloroform, or any other narcotic, you have simply paralysis from a sudden induction of an over percentage of the narcotic in your Anæsthetic. If you generate and introduce electricity in close observance, and in accordance with the laws of nature, your patient will receive it kindly, and you will not have fatal results. You commence the induction of electricity—after placing the poles of the battery in the hands of your patient—by turning on a very light volume, and then increase the volume as your patient is capable of receiving it. By so doing, you stimulate neural circulation *gradually*, and thereby increase the capacity to receive without producing concussion or paralyzation. In this way your patient is prepared to receive and retain a much greater volume of electricity than would have produced death had it been suddenly inducted. Fill a Leyden jar with this subtle agent and let it into a man all at once, and the functions of life will instantly cease. But you can fill fifty jars with electricity and turn it into him gradually on an insulated stool without injury. If he has a hat on his head it will likely tumble off, and his hair will stand up like the quills of a porcupine; but if he does not know what you are doing, he wonders that the hat and the hair behave so strangely, yet if it pass out of him gradually he is none the worse for it. Just so in generating and applying anæsthetics. If you generate and induct an over percentage of your narcotic element *suddenly*, especially if it be a powerful drug such as chloroform, you will likely produce a *shock* or concussion in the medulla of an extremely delicately, nervously constituted patient, in the first, second or third inhalation. This result is not produced by your narcotic through arterial and venous circulation, as is generally taught, but by the chemical action of the narcotic upon the nerve centre and through the sensory nerves. It is estimated that an ordinary sized man has about 1,400 square feet of surface in his lungs, over which the pneumo-gastric nerve, with its tens of thousands of nerve filaments are spread out, ready to apprehend and receive your anæsthetic as it is introduced. If your narcotic happens to be chloroform (for instance), of which it has been estimated by the Royal Medical and Chirurgical Society of England, that 3½ per cent. is sufficient for the average patient, 6 per cent. is dangerous and 12 per cent. is almost sure to produce asphyxia, plus-narcotism and may be death, and if your patient happens to be the delicate one previously described, and if you, instead of commencing the introduction of your anæsthetic with about one or two per cent. of the narcotic, *abruptly* commence with about 30 per cent., do not be surprised at your fatal result. You have violated nature's laws and must abide the consequences. You should be prosecuted for *mal-practice*, and suffer the penalty of your crime.

But if you commence the induction of your narcotic with about one per cent. and then increase the percentage just as your patient kindly receives and tolerates it, you will *never* produce concussion or paralyzation. But deaths sometimes occur in the latter stages of anæsthesia, and yet not without a cause. If you have a death from the secondary effect of your anæsthetic, it *is* produced by over-crowding the blood with the narcotic element, so as to crowd out the necessary quantity of free oxygen to furnish *vital force* sufficient to eliminate the narcotic and to sustain life. Therefore, when you have a death in what is known as the third stage of anæsthesia, you always have asphyxia first; and when you have it so profound, or have reduced the quantity of free oxygen in the blood, so that there is not sufficient *vital force* to eliminate the narcotic, your patient sinks and is soon irrevocably dead.

Hayes Process of Generating and Applying Anæsthetics, with the appropriate Apparatus removes the cause of these fatal results by regulating the pro rata or percentage of the narcotic in the anæsthetic, so as to not only know *positively* about how much of the narcotic is in the anæsthetic at every stage of anæsthesia, but to regulate the same so as to *adapt* it to the kindly tolerance of the patient in all the various stages of anæsthesia, and thus prevent a "*shock*" or concussion and paralyzation in the first stage of administration, and asphyxia and death in the latter stage. Nausea and vomiting are frequently unpleasant results in the administration of chloroform, ether, etc., by the ordinary methods of administering. This is caused first by the deposition of the globules of vapor into the buccal fluids. These also irritate the fauces, epiglottis, mucus membranes of the bronchial tubes and air cells of the lungs. Hence, if you *destroy* these globules of vapor, you will obviate these unpleasant results, which is done by the Hayes Process and Apparatus by friction in the production of counter currents of air within the Generator. Another frequent cause of nausea, is the sudden introduction of an *over-percentage* or undue quantity of the narcotic element, which produces *revulsion* in neural circulation in the sympathetic *pneumo-gastric* nerve in the stomach, very similar to that which is caused by violently striking this delicate organ. Therefore, if you will generate and apply your anæsthetic by destroying the globules of the vapor, and regulate the percentage of the narcotic element in the anæsthesic, so as to adopt it to the kindly tolerance of the patient in all the stages of anæsthesia, which is accomplished by the Hayes Process and Apparatus, you will neither have *nausea* nor *death* resulting from your anæsthetic when using a suitable narcotic as the subordinate element, Many physicians and dentists think an abnormal condition of the heart, or what is commonly known as organic heart disease, or inflamed bronchi or lungs, forbid the use of anæsthetics. This impression has been extended and confirmed by the notorious fact that the heart, the lungs and the brain have been made the *scape goat* for many a man's just punishment for his criminal mal-practice in the use of the so-called anæsthetics. When a death occurs a friend is sought to conduct an autopsy; he selects a few of his medical friends, whom he can readily influence, and they proceed to investigate the heart, the lungs and the brain, and when through with the *post mortem* examination, they announce that they found clots of blood in the heart, and that there was evident valvular disease of that organ; or that they found a congested condition of the brain, the arteries contracted and evidences of embolism, and that the death occurred from the diseased condition of these organs. Now, I submit to your intelligence, whether even alcohol, much more sulphuric ether, chloroform, etc., will not produce a congested condition of the brain, contract the arteries, and produce evidence of embolism, and whether you are not likely to find even in the heart of a slaughtered beef, clotted blood, much more so would you find clots of blood in the heart of any animal from whom the blood has not been previously taken. Such autopsies may suffice to shield the character and reputation of the perpetrator, or the man himself from his just punishment for mal-practice, but I protest against its being received as a scientific investigation of cause and effect. If the heart is diseased what is the cause of the specific disease? If the left or right mitral valve drops strokes, we find that there is a remittant flow of *nervo-vita* fluid in the moter nerves of these valves. If there is dilitation or fatty degeneracy of the heart, it can invariably be traced to an imperfect circulation of the nervo-vital fluid in the nerves of the muscles of the heart. But if you induct your anæsthetic in the manner here described, you will not interrupt neural circulation, and the functions of life, will be in continuance, and you have anæsthesia safely produced.—*D. & S. Microcosm.*

